# Oncogenic Deregulation of Cell Adhesion Molecules in Leukemia

**DOI:** 10.3390/cancers11030311

**Published:** 2019-03-05

**Authors:** Roland Windisch, Nina Pirschtat, Christian Kellner, Linping Chen-Wichmann, Jörn Lausen, Andreas Humpe, Daniela S. Krause, Christian Wichmann

**Affiliations:** 1 Department of Transfusion Medicine, Cell Therapeutics and Hemostaseology, University Hospital, LMU Munich, 81377 Munich, Germany; Roland.Windisch@med.uni-muenchen.de (R.W.); n.pirschtat@gmx.de (N.P.); Christian.Kellner@med.uni-muenchen.de (C.K.); Linping.Chen-Wichmann@med.uni-muenchen.de (L.C.-W.); Andreas.Humpe@med.uni-muenchen.de (A.H.); 2 Institute for Transfusion Medicine and Immunohematology, Johann-Wolfgang-Goethe University and German Red Cross Blood Service, 60528 Frankfurt am Main, Germany; lausenj@gmail.com; 3 Institute for Tumor Biology and Experimental Therapy, Georg-Speyer-Haus, 60596 Frankfurt am Main, Germany; Krause@gsh.uni-frankfurt.de

**Keywords:** adhesion, leukemia, oncogene, integrin, CAM

## Abstract

Numerous cell–cell and cell–matrix interactions within the bone marrow microenvironment enable the controlled lifelong self-renewal and progeny of hematopoietic stem and progenitor cells (HSPCs). On the cellular level, this highly mutual interaction is granted by cell adhesion molecules (CAMs) integrating differentiation, proliferation, and pro-survival signals from the surrounding microenvironment to the inner cell. However, cell–cell and cell–matrix interactions are also critically involved during malignant transformation of hematopoietic stem/progenitor cells. It has become increasingly apparent that leukemia-associated gene products, such as activated tyrosine kinases and fusion proteins resulting from chromosomal translocations, directly regulate the activation status of adhesion molecules, thereby directing the leukemic phenotype. These observations imply that interference with adhesion molecule function represents a promising treatment strategy to target pre-leukemic and leukemic lesions within the bone marrow niche. Focusing on myeloid leukemia, we provide a current overview of the mechanisms by which leukemogenic gene products hijack control of cellular adhesion to subsequently disturb normal hematopoiesis and promote leukemia development.

## 1. Introduction

### 1.1. Cell Adhesion Molecule Families

**Figure 1:**
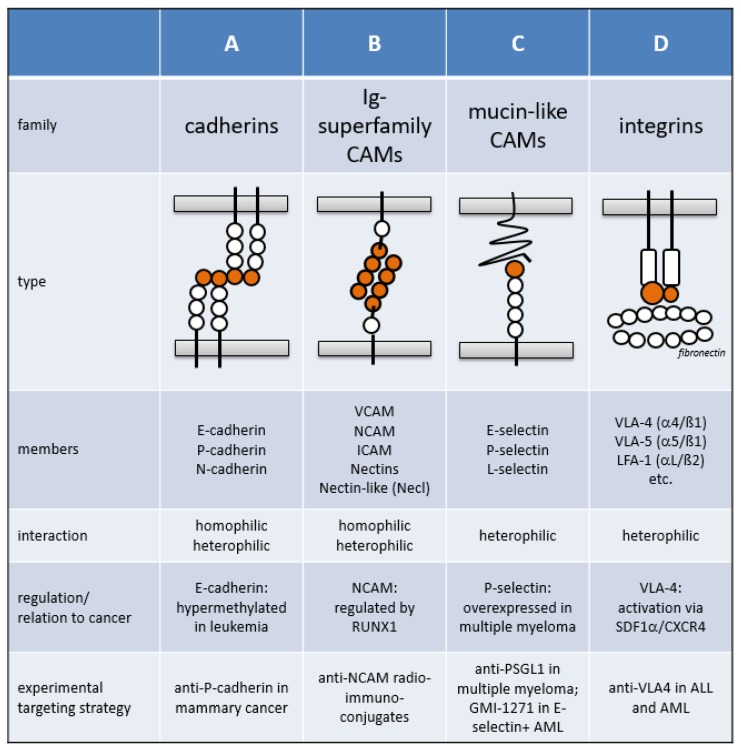
Structural and functional properties of the four major cell adhesion molecule families.

Cellular adhesion plays a vital role in many physiological and pathological processes, including developmental morphogenesis, differentiation, inflammatory responses, angiogenesis, wound healing, tumor progression, and metastasis [1]. Cellular adhesion is mediated by CAMs, which are found in metazoans ranging from sponges to complex mammals. CAMs also further modulate intracellular signal transduction, thereby regulating self-renewal, differentiation, and apoptosis. The three conserved CAM domains include an intracellular domain (which interacts with the cytoskeleton), a transmembrane domain, and an extracellular domain. Junctional adhesion mechanisms, which mainly grant intercellular tight junctions in cell assemblies, are distinct from non-junctional adhesion mechanisms, such as integrin- and selectin-mediated adhesion. Based on the protein sequences and structures, CAMs have been classified into four major types: the cadherin superfamily, the immunoglobulin superfamily (IgCAM), selectins, and integrins (Figure 1).

#### 1.1.1. The Cadherin Superfamily

The cadherin superfamily, named for “calcium-dependent adhesion”, includes cadherins, protocadherins, desmogleins, and desmocollins. Structurally, all types share the so-called cadherin repeats, an extracellular Ca^2+^-binding domain. Cadherins grant the maintenance of adult tissue architecture and play an important role in junctional adhesion to bind cells within tissues together. Structurally, cadherins fold homophilic interactions, although heterotypic binding has also been observed [2]. The various classes of cadherin molecules are associated with different tissue types: epithelial (E)-cadherin, placental (P)-cadherin, and neural (N)-cadherin [3]. Cells containing the same cadherin tissue subtype tend to cluster together, both in cell culture and during development. Disruption of cadherin-mediated cell–cell adhesion is a common feature of several solid and hematological tumor types. P-cadherin seems to play an important role in solid cancer progression and metastasis and thus is considered as a promising therapeutic antibody target [4]. The E-cadherin locus has been suggested as a common target for hypermethylation in leukemia [5,6]. Low expression of E-cadherin has been found in acute myeloid leukemia (AML) and chronic myeloid leukemia (CML) cells and is associated with loss of adhesion and density-dependent contact inhibition, thus promoting cell growth [7]. However, in erythroid leukemia, E-cadherin is expressed in myeloid blasts and displays a specific cell surface marker signature including CD34+ and CD117+ [8]. N-cadherin appears to play a crucial role in sustaining leukemic stem cell self-renewal, thus further evading the effects of chemotherapy. Etoposide-treated human KG1a AML cells grown on N-cadherin-Fc chimera-coated plates have shown increased IC_50_ compared with controls. Furthermore, transplantation of the N-cadherin-positive CD34+/CD38− cell fraction from AML samples into NOD/SCID mice has revealed stronger leukemic engraftment and enhanced self-renewal capacity compared with N-cadherin-negative control cells [9]. Moreover, upregulation of N-cadherin expression levels has been found in t(8;21)+ AML-M2 samples, with increased self-renewal properties of leukemic progenitor cells [10].

#### 1.1.2. The Immunoglobulin Superfamily

Immunoglobulin family members on neighboring cells form homophilic and heterophilic interactions by binding similar molecules and typically function independent of calcium. Members include vascular cell adhesion molecules (VCAMs), intercellular adhesion molecules (ICAMs), neural cell adhesion molecules (NCAMs), nectins, and nectin-like (Necl) proteins [11]. VCAM-1 is expressed on activated endothelial cells, where it binds integrins α4/β1 (very late antigen-4, VLA-4) and α4/β7 (lymphocyte Peyer’s patch adhesion molecule 1, LPAM-1) and regulates adhesion of lymphocytes and myeloid cells to the endothelium of inflammatory sites. *VCAM-1* expression is induced by cytokines, such as IL-1 and TNFα, and endotoxins, such as LPS. VCAM-1 mediates leukocyte endothelial cell signal transduction and may be involved in atherosclerosis and rheumatoid arthritis [12].

ICAMs direct activated leukocytes to areas of tissue damage. These molecules are induced by cytokines such as interferon-γ, IL-1, and TNFα, which are secreted after injury. Although ICAMs are primarily expressed by immune and endothelial cells, brain-specific forms also exist. In a recent study, Liu et al. have found that ICAM-1 plays a critical role in maintaining quiescence of hematopoietic progenitor cells in the bone marrow niche [13]. All ICAMs share the counter receptor lymphocyte function-associated antigen-1 (LFA-1, CD11a/CD18, αL/β2 integrin). LFA-1 expressed on the surface of leukocytes modulates adhesion-dependent events that are essential for immune system activity such as immune synapse formation. In the brain, *LFA-1* expression, which is linked to microglia activation, is restricted to resident macrophages and microglia. ICAM-1 has been suggested as a target antigen for therapeutic antibodies to treat multiple myeloma and other cancers [14,15,16,17]. However, in a phase II trial in patients with smoldering multiple myeloma, no relevant efficacy was observed [18].

NCAM (CD56) is another glycoprotein of the Ig superfamily. At least 27 alternatively spliced *NCAM* mRNAs yield a wide diversity of NCAM isoforms. NCAM is expressed on the surface of neurons, glia cells, skeletal muscle cells, and certain leukocytes such as natural killer cells. Homophilic NCAM binding provokes the activation of signaling cascades leading to cellular responses like survival or differentiation. Moreover, heterophilic interaction with extracellular proteins such as fibroblast growth factor receptor (FGFR) seems to play a role in promoting neurite outgrowth [19].

Nectins and Necl molecules are expressed in a number of cell types, in which they are important for cell–cell adhesion and the formation of stable adherens junctions via homophilic (in cis) and heterophilic (in trans) auto-interactions [20]. These molecules play a role in various cellular activities including cell polarization, cell migration, cell growth, and cell fate. Nectins and Necls interact with and share a number of binding partners through their cytoplasmic domain. However, only nectins bind to intracellular afadin, an F-actin binding protein. In particular, nectins are involved in the formation of cadherin-based cell–cell junctions, mediating initial cell–cell contacts via nectin–nectin or nectin–Necl binding and establishing links to the actin cytoskeleton via nectin–afadin binding [21,22], thus indicating that they may also be involved in cell migration [23]. Certain nectins and Necls have also been proposed to play an important role in cancer immune surveillance. For example, the nectins polio-virus receptor (PVR, CD155) and CD112 have been suggested to modulate cytotoxic lymphocyte responses through binding to the activating and adhesion receptor DNAX accessory molecule-1 (DNAM-1, CD226) as well as inhibitory receptors, T-cell immunoreceptor with Ig and ITIM domains (TIGIT), and CD96. Interestingly, antibody blockade of these nectins enhanced T-cell-mediated killing of AML cells in vitro. Moreover, in AML patients, high expression levels of *PVR* and *CD112* correlated with poor prognosis, thus suggesting that CD155 and CD112 or their cognate receptors may be targeted in cancer immune checkpoint antibody therapy [24,25].

#### 1.1.3. Mucin-like CAMs

Selectins are a group of CAMs involved in lymphocyte homing, atherosclerosis, lupus erythematosus, cancer metastasis, and acute and chronic inflammation in kidney, muscle, heart, and skin [26]. Selectins are single-chain transmembrane glycoproteins, which bind sugar polymers in a calcium-dependent manner. The three subsets of selectins display distinct expression patterns: E-selectin is mainly expressed on endothelial cells, L-selectin is expressed on leucocytes, and P-selectin is primarily expressed on platelets and endothelial cells. P-selectin is stored intracellularly in storage granules, which enables rapid activation [27,28], E-selectin requires de novo transcription, and L-selectin is constitutively expressed on almost all leukocyte subpopulations.

P-selectin glycoprotein ligand-1 (PSGL-1, SELPLG, or CD162), the best-characterized ligand of the three selectins, is a 220-kDa disulfide-linked homodimeric sialomucin expressed on activated endothelial cells, myeloid cells, and lymphoid cells. PSGL-1 plays a critical role in tethering white blood cells to activated platelets or endothelia-expressing P-selectin. PSGL-1 binds all three members of the selectin family, although it displays highest affinity to P-selectin. White blood cells do not typically interact with the endothelium of blood vessels. However, inflammation causes the expression of P-selectin on the blood vessel wall [29]. Tethering is initiated when PSGL-1 interacts with P-selectin and/or E-selectin on endothelial cells and adherent platelets. This interaction induces rolling on the endothelial cell surface and subsequent stable adhesion and transmigration of the white blood cell into the inflamed tissue [30]. PSGL-1 also harbors binding sites for the chemokines CCL19 and CCL21 and efficiently regulates homing of T-cells to secondary lymphoid organs [31].

Selectins also play an important role in cancer progression and metastasis in particular [26,32]. For example, selectin ligands are highly expressed on endothelial vessels at the site of tumor metastasis, thereby yielding a potential entry site for circulating tumor cells [33]. Additionally, E-selectin, the glycoprotein to which leukemia-initiating cells adhere [34], is upregulated in the bone marrow vasculature in leukemia, thereby encouraging survival signaling and provoking chemoresistance via binding to PSGL-1 and CD44. In mice, xenotransplanted *CD44*^−/−^*PSGL-1*^−/−^ AML cells were 100-times more sensitive to high-dose cytarabine chemotherapy than wild-type AML control cells. While the E-selectin antagonist GMI-1271 has been shown to enhance chemosensitivity to cytarabine in control mice, enhanced chemosensitivity was not observed in mice transplanted with *CD44*^−/−^*PSGL-1*^−/−^ AML cells. Importantly, E-selectin inhibition has resulted in downregulation of components of the PI3K/AKT/NF-κB pathway. Downregulation was due to a lack of phosphorylated AKT(Ser^473^) and NF-κB/p65(Ser^536^), which rapidly takes place after adhesion to E-selectin by AML blasts in vitro, thereby suggesting that AKT phosphorylation may be a potential mechanism to promote chemoresistance [35]. E-selectin is upregulated via activated STAT3 in endothelial cells, thereby triggering adhesion and tumor cell invasion, as recently demonstrated [36]. Furthermore, monocytes attracted to cancerous lesions also induce E-selectin through secretion of soluble CCL2, thereby promoting tumor cell metastasis [37].

#### 1.1.4. The Integrin Family

Integrins comprise a large and complex family of transmembrane glycoproteins. These molecules transmit signals via inside-out and outside-in signaling. Integrins are composed of alpha- and beta-chains that form non-covalent heterodimers [38]. Currently, 18 alpha-subunits, 8 beta-subunits, and 24 unique heterodimers have been described. Integrins are widely expressed in many tissues, including hematopoietic cells, and form heterophilic interactions by binding various members of the CAM family. They are crucial for the interaction of hematopoietic cells with stromal cells and secreted or insoluble components of the extracellular matrix. Integrins, such as VLA-4 (α4/β1), play an essential role in several cellular processes, including homing of bone marrow-derived blood cells to various organs, controlling cellular shape and movement, cell proliferation, and lineage commitment. *VLA-4* is typically expressed on leukocytes including monocytes, lymphocytes, eosinophils, basophils, and CD34+ hematopoietic precursor cells. *VLA-4* expression on CD34+ cells is upregulated by the cytokines interleukin-3 (IL-3) and stem cell factor (SCF). By contrast, *VLA-4* is downregulated by granulocytic colony stimulating factor (G-CSF). As G-CSF mobilizes CD34+ cells from the bone marrow, it is used in peripheral blood cell apheresis for autologous and allogenic stem cell transplantation [39]. In the bone marrow, integrins are further required for the proper interaction of stem and progenitor cells with molecules of the extracellular niche matrix, such as fibronectin or periostin, to ensure lifelong self-renewal. Expression and activation of integrins therefore require careful regulation. As these processes are actively abused by cancer cells, integrins also seem to be involved in malignant cell growth and metastasis [40] and have therefore been evaluated as targets for cancer therapy [41,42].

### 1.2. Adhesion Molecules of the Hematopoietic Stem Cell Niche

**Figure 2:**
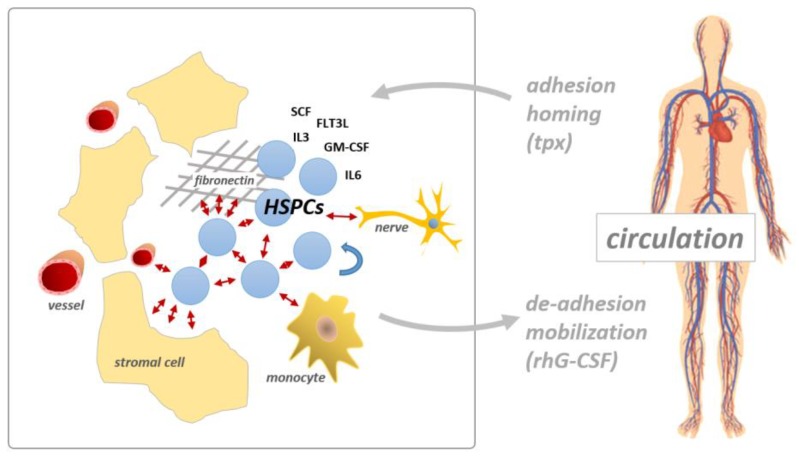
Adhesion molecule-mediated cell–cell interactions in the hematopoietic stem cell niche. Hematopoietic stem and progenitor cells (HSPCs) are able to assemble homotypic and heterotypic cell–cell and cell–matrix interactions (red arrows), via cell adhesion molecules (CAMs), such as VLA-4, VLA-5 and LFA-1, thereby regulating proliferation, self-renewal and differentiation. tpx, transplantation; rhG-CSF, recombinant human G-CSF.

Hematopoiesis involves a tightly regulated interaction between HSPCs and other cell types including sinusoidal endothelial cells, mesenchymal stromal cells (MSCs), osteoblasts, macrophages, and sympathetic nerve fibers in the HSC niche [43,44,45,46]. The HSC niche regulates the proliferation, self-renewal, and differentiation of HSCs by producing positive and negative bi-directional cues, which are induced by adhesion molecule-mediated cell–cell and cell–matrix interactions [47,48]. A schematic illustration of the HSPC homotypic and heterotypic interactions in the HSC niche is presented in Figure 2. HSPCs express numerous cell adhesion molecules, including integrins VLA-4 (α4/β1, a receptor of VCAM-1 and fibronectin), VLA-5 (α5/β1, a receptor of fibronectin), and LFA-1 (αL/β2, a receptor of ICAM-1), which are also expressed on both hematopoietic and stromal cells. HSPCs also express the c-KIT receptor tyrosine kinase, which both exerts kinase activity and functions as an adhesion molecule, as the receptor binds cell surface-bound SCF. Of note, c-KIT-mediated niche anchorage is kinase independent and does not respond to the kinase inhibitor imatinib [49]. Additionally, PSGL-1, N-cadherin, and glycoprotein CD44, which is recognized by hyaluronan, osteopontin (secreted phosphoprotein 1, SPP1), and E-selectin, are all expressed on HSPCs [50,51]. The interactions between the ß1 integrins VLA-4 and VLA-5 expressed on HSPCs and the bone marrow niche are important to maintain steady state hematopoiesis. For example, the interaction between VLA-4 and fibronectin is essential for both maintaining HSC self-renewal capacity and regulating HSC differentiation. Interestingly, administering VLA-4-specific antibodies augments the G-CSF-induced mobilization of long-term repopulating HSCs from the bone marrow into the peripheral blood [52,53,54]. VLA-4 may also play a role in chemotherapy resistance, functioning presumably via pro-survival signals to leukemia cells [55]. Similarly, VLA-5 is important for HSPC homing and proliferation [56]. The understanding of the molecular mechanisms of HSC anchorage in the bone marrow niche has led to the development of several inhibitors targeting critical adhesion molecules, such as VLA-4, PSGL-1, and CXCR4, to mobilize CD34+ hematopoietic progenitors [57]. These inhibitors enable the collection of HSPCs via apheresis techniques for autologous and allogeneic stem cell transplantation [58,59]. Furthermore, targeting adhesion may represent an additional therapeutic option to clear the bone marrow stem cell niche of resistant leukemia cells.

### 1.3. Oncogenic Lesions in Myeloid Leukemia

Mutations of proto-oncogenes, which are typically dominant in nature, cause normal cells to become cancerous. Proto-oncogenes encode proteins that stimulate cell division, regulate self-renewal, inhibit differentiation, and impede cell death. Each of these processes is important for normal cellular development and maintenance of tissues and organs. Oncogenes, however, typically exhibit increased protein production or activity, thereby prompting malignant growth [60]. As a result, oncogenes represent major targets in drug design [61]. In myeloid leukemia, two major classes of oncogenic events have been described, namely, chromosomal translocations resulting in novel fusion proteins and activated receptor tyrosine kinases [62]. Chromosomal translocations often involve transcription factors that normally regulate cellular differentiation. The translocation product then disrupts normal transcription factor function leading to a block in cellular differentiation and enhanced self-renewal. By contrast, activating mutations in receptor tyrosine kinases disrupt cellular signaling leading to enhanced cell growth and evasion of apoptosis. Results from the last decades uncovered an evolutionary leukemia model in which chromosomal translocation products and hyperactivated kinases play a critical role in a stepwise transformation process [63,64]. The mutational oncogenic activation mechanisms of CAMs have not yet been described. Compared with genes such as *c-KIT* and *FLT3* (FMS-like tyrosine kinase 3), CAMs are much less frequently found to be mutated in cancer cells (Table 1). However, adhesion molecule expression and function are frequently deregulated in leukemia. Oncogene-mediated activation of cell adhesion affects interactions within the stem cell niche and within other tissues. The latter aspect may account for the complications associated with leukostasis including leukemic meningitis, leukemia cutis, extra-medullary leukemia, and formation of myeloid sarcoma. In the following chapter, we summarize recent findings concerning the mechanisms by which leukemogenic gene products control adhesion and migration and discuss the consequences of direct CAM regulation on leukemia development.

## 2. Oncogenes Directly Deregulate Cellular Adhesion

Distinct oncogenes are closely associated with specific leukemia subtypes. The molecular pathology of CML was elucidated by the discovery of the recurrent chromosomal translocation t(9;22) that results in the expression of the hyperactive tyrosine kinase BCR-ABL1. BCR-ABL1 triggers various intracellular signal transduction pathways including JAK/STAT, PI3K, and RAS/RAF, thereby supporting leukemic cell growth [65]. Intriguingly, BCR-ABL1 also influences cell adhesion molecule function. In retrovirally transduced BCR-ABL1-overexpressing hematopoietic bone marrow progenitor cells, expression levels of PSGL-1 and L-selectin were diminished compared with control cells, while other adhesion molecules remained unaffected [66] or even were overexpressed [67]. As a result, BCR-ABL1-positive murine progenitors showed poor engraftment after intravenous injection into irradiated syngenic recipient mice. Notably, E-selectin, but not P-selectin, is critically required for homing and engraftment of BCR-ABL1+ leukemia-initiating cells. Indeed, engraftment of BCR-ABL1+ leukemia-initiating cells has been shown to be reduced by antibody-mediated selectin blockade. Therefore, despite the significant therapeutic effects of tyrosine kinase inhibitors (TKIs), the authors suggested that TKI-refractory CML patients undergoing autologous stem cell transplantation may benefit from anti-E-selectin treatment to block engraftment of leukemia-initiating cells persisting in the autologous HSC graft, while favoring engraftment of “healthy” CD34+ counterparts. Accordingly, the combination of TKI, imatinib, and the E-selectin inhibitor, GMI-1271, reduced leukemia engraftment and prolonged the survival of mice transplanted with CML-initiating cells in a murine model. This observation could be explained by enhanced cell cycle activity and increased expression of the transcription factor TAL1 upon treatment with GMI-1271. Importantly, the TAL1 transcription factor negatively regulates CD44 expression, a ligand of E-selectin. Mechanistically, TAL1 activity was further enhanced by imatinib via the reduction of AKT-mediated inhibitory phosphorylation. Hence, both GMI-1271 and imatinib display promising therapeutic potential to eradicate leukemic CML stem cells [51]. Together, these data do not support the concept that BCR-ABL1-mediated selectin deregulation represents a disease progression factor. However, a former study has shown that VLA4 and VLA5, which mediate cell adhesion to extracellular matrix ligands, were positively affected by BCR-ABL1 [68]. In the presence of the cytokines GM-CSF and IL-3, binding of BCR-ABL1+ cells to fibronectin and ICAM-1 was enhanced. BCR-ABL1-stimulated integrin activation and increased ligand binding to fibronectin and ICAM-1 were shown to be tyrosine kinase-dependent. However, the exact mechanism of VLA-4 and VLA-5 activation remains unclear. Consistent with these results, hematopoietic precursor cells from CML patients have shown increased adhesion to fibronectin.

The reciprocal fusion protein ABL1-BCR, an N-terminally truncated BCR mutant, lacks fundamental functional features of BCR regarding regulation of small Rho-like GTPases [69]. These GTPases are critical intracellular transducers of cellular adhesion programs. The Rho-GEF and Rac-GAP functions of BCR strongly suggest that BCR-ABL1 plays an important role in cytoskeleton modeling by regulating Rho-like GTPases, such as RHO, RAC, and CDC42 [70]. However, expression of the reciprocal ABL1-BCR displayed negative effects on cell motility, in particular involving the capacity to adhere to and transmigrate through the endothelial cell “barrier”. This observation suggests a further retention mechanism of t(9;22)+ leukemia cells within the bone marrow stroma. The exact function of ABL1-BCR in cellular adhesion needs to be clarified in the context of BCR-ABL1 in a cellular setting with simultaneous expression of both oncogenes.

Activating mutations of the *FLT3* gene represent one of the most frequently encountered and clinically challenging classes of AML mutations [71,72], which may be due to insufficient eradication of slow cycling leukemia stem cells within the bone marrow microenvironment. FLT3-ITD, an internal tandem duplication of *FLT3*, also activates VLA-4-dependent adhesion of hematopoietic progenitor cell lines to stromal cells [73,74]. In this study, FLT3-ITD triggered phosphorylation and activation of PYK2, a member of the focal adhesion kinase (FAK) family that is highly expressed in hematopoietic cells. PYK2 is involved in signal transduction nodes of the intracellular integrin signaling pathway. Notably, PYK2-triggered VLA-4 activation is independent of RAP1, a critical positive regulator of inside-out integrin signaling. Intriguingly, FLT3-ITD has been found in a multi-protein complex together with PYK2 and the ß1 integrin subunit of VLA-4. Via direct regulation of VLA-4, FLT3-ITD bypasses normal inside-out signaling required for VLA-4 activation. The authors have stated that VLA-4 activation by FLT3-ITD might contribute to FLT3-ITD-induced AML development with poor drug responsiveness and prognosis of this leukemia entity. FLT3-ITD+ blast cells attach to the niche, via VLA-4/VCAM-1, and receive pro-survival signals. The authors further proposed FLT3 small molecule inhibitors as anti-adhesive therapy for AML patients with activated FLT3. Inhibition of VLA-4-mediated attachment to the bone marrow niche may decrease survival and anti-proliferative signals from niche cells, thereby rendering leukemia cells more sensitive to chemotherapeutic drugs. Recently, integrin β3 has been shown to be substantially involved in leukemogenesis and leukemic stem cell maintenance. Thus, in AML, integrin αvβ3 is able to enhance β-catenin activation through PI3K/AKT/GSK3β signaling and therefore constitutes an important prognostic marker, especially for patients with the FLT3-ITD mutation. In addition to activation via αvβ3, the FLT3-ITD mutation causes an increase in the activated form of β-catenin. Therefore, patients with FLT3-ITD mutation may benefit from combined therapy consisting of TKI, αvβ3 blocker, and β-catenin inhibitor [75,76]. Moreover, FLT3 has been suggested as a target for antibody therapy for AML patients, and such antibodies in particular may be promising in combination with TKIs, which upregulate cell surface FLT3 expression levels [77]. The recently discovered αvβ3 addiction of MLL-AF9 leukemia may also be FLT3-dependent [78].

A related tyrosine kinase, JAK2, is frequently mutated in polycythemia vera (PV), which is a myeloproliferative neoplasm in which the bone marrow overproduces red blood cells. The disease may also result in the overproduction of white blood cells and platelets. The main health concerns of PV are caused by increased blood viscosity as a result of the increased hematocrit. Activating mutations of JAK2 have been identified as strong driver oncogenes in this disease entity [79,80,81,82,83]. The activated tyrosine kinase JAK2V617F induces adhesion of PV red blood cells to laminin, a ubiquitously expressed glycoprotein also found on endothelial cells [84,85]. This interaction is dependent on the Lutheran (Lu) blood group/basal cell-adhesion molecule (Lu/BCAM) expressed on PV cells. JAK2V617F induced activation of the RAP1/AKT pathway, independent of the erythropoietin (EPO) receptor pathway. AKT then triggers Lu/BCAM phosphorylation, thereby enabling adhesion to laminin on endothelial cells. This cell–cell interaction between circulating PV red blood cells and endothelial cells may represent the primary event in PV-associated thrombosis, a major clinical complication of PV [86]. Notably, the blood group antigen Lu/BCAM is also involved in other diseases including sickle cell disease [87].

CEBPα, one of the key transcription factors deregulated in AML [88], regulates cellular adhesion via the stem cell homing receptor CXCR4 [89]. CXCR4 is an alpha-chemokine receptor specific for SDF1α, a chemokine important for HSC homing to the bone marrow, retention of HSCs in the bone marrow, and HSC quiescence. CXCR4 is critically involved in the cross-talk between leukemia cells and the bone marrow microenvironment and has been suggested as a worthwhile target to eradicate leukemia. Using immune deficient mouse models, CXCR4 antagonists were found to mobilize human AML cells into the circulation and enhance anti-leukemic effects of chemotherapy in vivo [57,90]. In AML patients, high *CXCR4* expression levels are generally associated with poor prognosis, independent of the *FLT3* mutational status [91]. Kuo et al. have found that wild-type CEBPα upregulates *CXCR4* expression in hematopoietic cell lines [89]. Chromatin immunoprecipitation experiments have revealed that CEBPα directly binds to the *CXCR4* promoter region, thereby transactivating *CXCR4* gene expression. Notably, the N-terminal CEBPα mutant in AML is unable to transactivate CXCR4. The CEBPα mutant did not show a dominant negative effect over wild-type CEBPα, and only patients with a bi-allelic *CEBPα* mutation displayed better prognosis after chemotherapy [92]. Even with high SDF1α concentrations, the absence of CXCR4 leads to detachment from the microenvironment. Once detached, the cells lose the capacity for self-renewal, quiescence, and protection against chemotherapeutic drugs. As CXCR4 is also critically involved in integrin VLA-4 activation, the cells further lose VLA-4-mediated protection from chemotherapeutic drugs [55]. This may at least in part represent the molecular basis for the observed favorable prognosis in CEBPα double-mutant AML patients [93].

**Figure 3:**
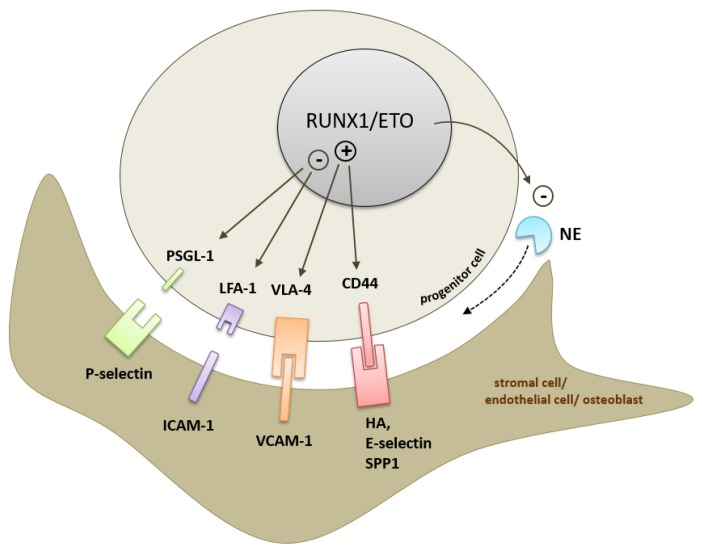
RUNX1/ETO-induced CAM signature within the bone marrow microenvironment. The cell adhesion molecules PSGL-1, LFA-1, CD44, and VLA-4 have been described as target genes directly regulated by RUNX1/ETO [100,104,105,107]. Neutrophil elastase (NE) has been described as a RUNX1/ETO-repressed target gene [114].

RUNX1, a further transcription factor frequently mutated in AML, directly regulates the immunoglobulin member *NCAM* [94,95]. *NCAM* is expressed in subtype M2 and M5 AML cells and correlates with poor overall survival [96,97]. Interestingly, wild-type RUNX1 upregulates *NCAM*, whereas shorter RUNX1 isoforms repress *NCAM* transcription. So far, NCAM has been targeted in antibody therapy to treat metastasized neuroblastoma employing radio-immunoconjugates [98,99]. Adhesion molecule deregulation has also been reported for the chimeric *RUNX1/ETO* (also known as *AML1/ETO*) fusion gene. Ponnusamy et al. have shown that α4 and β1 integrin subunits, together forming integrin VLA-4, are transcriptionally upregulated in t(8;21)+ AML cells expressing the *RUNX1/ETO* fusion gene [100]. RUNX1/ETO is a constitutive transcriptional repressor of RUNX1 target genes, thereby deregulating numerous genes involved in blood cell development [101]. The fusion gene has further been shown to play a role in transcriptional activation [102,103]. RUNX1/ETO has also been shown to regulate genes of the adhesion molecule family, such as *CD44* and *LFA-1* [104,105]. The degree of RUNX1/ETO-mediated α4 and β1 upregulation directly correlates with oncogene expression levels. ShRNA- and peptide-based RUNX1/ETO inhibition has revealed that expression of *VLA-4* remained dependent on RUNX1/ETO in human transformed t(8;21)+ Kasumi-1 cells. Furthermore, RUNX1/ETO has been shown to activate the VLA-4 integrin under shear stress and induce VLA-4-dependent migration of hematopoietic progenitors towards stromal-derived-factor-1 (SDF-1α, CXCL12), a chemokine highly concentrated in the stem cell niche. Notably, a splice-variant of RUNX1/ETO with increased leukemogenic potential [106] has been found to upregulate α4- and β1-integrin subunits more efficiently than full-length RUNX1/ETO. Interestingly, the occurrence of the splice-variant also correlated with poor prognosis and a more immature leukemia cell phenotype, thus suggesting that VLA-4-dependent integrin signaling directly influences the t(8;21)+ AML phenotype. Together, the data reveal a direct link between RUNX1/ETO and VLA-4 integrin-mediated adhesion/migration of hematopoietic progenitor cells. RUNX1/ETO also directly regulates the adhesion family gene *PSGL-1*, in which the fusion protein acts as a transcriptional repressor by epigenetically silencing the gene via binding to the *PSGL-1* promoter in hematopoietic progenitor cells. Importantly, studies have shown that a DNA-binding defective mutant was not able to regulate *PSGL-1* expression. Disruption of PSGL-1-mediated cellular adhesion was fully restored by depletion or inhibition of RUNX1/ETO, as the remaining RUNX1 is able to bind to the promoter in the absence of the oncogene leading to *PSGL-1* re-expression. Consequently, RUNX1/ETO-depleted cells regained their potential to bind to P-selectin and E-selectin coated beads [107]. Further studies have shown that RUNX1/ETO exerts a more profound effect on the regulation of genes involved in migration and cell-to-cell adhesion. While *RUNX1/ETO*-overexpressing cells have shown increased cell motility and migration, a reduction in cell-to-stroma adhesion has been demonstrated in vitro. Additionally, *RUNX1/ETO* expression impairs HSPC homing and engraftment, thus suggesting an abnormal interaction with the hematopoietic niche [108]. In summary, the RUNX1/ETO-induced CAM signature is depicted in Figure 3.

Adhesion molecule deregulation has also been reported for other fusion genes resulting from chromosomal translocation. For example, afadin (AF6), the complex partner of Nectin that normally controls integrin-mediated adhesion, is a common translocation partner in mixed lineage leukemia [109,110,111]. MLL-AF6 has been shown to sequester AF6 into the nucleus, thereby leading to aberrant RAS activation [112]. As loss of AF6 enhances migration and invasion of tumor cells [113], MLL-AF6 may also affect cell adhesion. However, the distinct role that MLL-AF6 plays in adhesion and migration has not been described.

While the mechanism has not yet been deciphered, upregulation of the sialomucin PSGL-1 is critically involved in the development and drug responsiveness of multiple myeloma, an adjacent hematological malignancy with lymphatic origin [115]. Multiple myeloma mutations are recurrently found in *N-RAS* and *K-RAS* [116]. However, RAS is not a direct regulator of PSGL-1. Potential regulators of PSGL-1 include STAT1, c-JUN, and NF-κB (UCSC Genome Browser), which are activated during immune response. The myeloma cells contact endothelial cells and stromal cells via PSGL-1 within the bone marrow niche. Using loss-of-function studies and the small molecule pan-selectin inhibitor GMI-1070, Azab et al. have shown that PSGL-1 activates β1-integrin and its downstream targets FAK, Src, cofilin, AKT, and GSK-3α/β [115]. Inhibiting this interaction using GMI-1070 enhanced the sensitization of multiple myeloma cells to the proteasome inhibitor bortezomib. This might represent the basis for targeted treatment of multiple myeloma patients with PSGL-1 neutralizing antibodies and/or GMI-1070 [117,118,119]. An alternative approach to block PSGL-1 function includes targeting the sialyltransferase ST3GAL6, which plays a key role in selectin ligand synthesis [66,120].

Taking a look beyond the transformed blast cells, a recent report has suggested that genetic aberrations in mesenchymal stromal cells (MSCs) also contribute to leukemia development and response to chemotherapy treatment [121]. Blau et al. have analyzed genetic aberrations in leukemic blasts and bone marrow-derived MSCs isolated from AML and MDS patients. Compared to non-mutated MSCs, patients harboring MSC aberrations displayed a worse clinical outcome in the group of AMLs with abnormal karyotype. As the microenvironment supports MDS and AML clones as a result of reciprocal interactions, abnormalities in MSCs might worsen those abnormalities intrinsic to the neoplastic cells. These aberrations might target adhesion molecule activity in MSCs, which then trigger leukemic blast cell growth and/or survival. This was the first suggestion that mutations in bone marrow niche cells also contribute to the survival of leukemia patients. Overall, adhesion molecules represent highly attractive therapeutic targets during the course of leukemia development.

## 3. Summary and Conclusions

**Figure 4:**
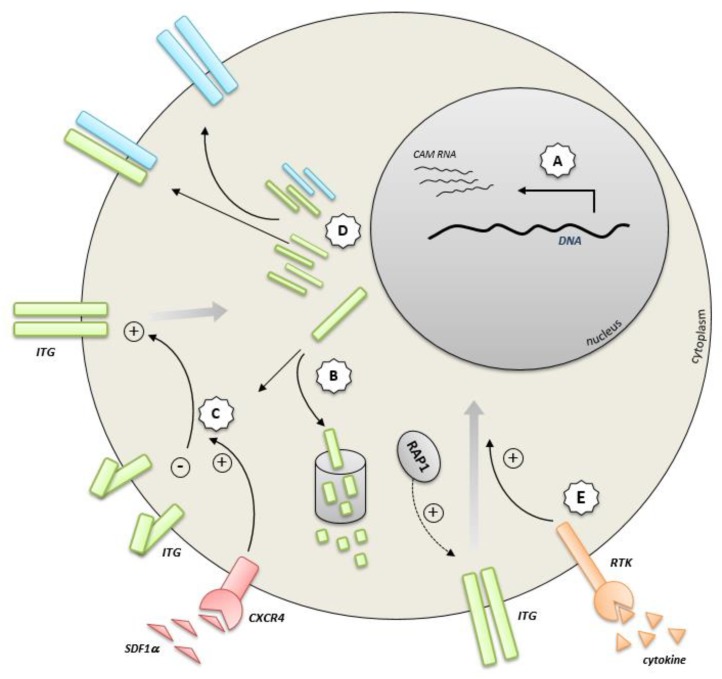
Described and hypothesized mechanisms of oncogenic interference with adhesion molecule function. (**A**) Transcriptional deregulation of adhesion molecule expression by aberrant transcription factors (e.g., RUNX1/ETO [100] or TAL1) [51]. (**B**) Interference with cellular destruction of adhesion molecules through proteasomal degradation. (**C**) Activation of integrin receptors by co-receptors (e.g., the SDF1α/CXCR4/VLA4 axis) and modulators thereof (e.g., CEBPα) [89]. (**D**) Interference with the composition of the adhesion molecule complex. (**E**) Superactivation of pre-activated adhesion molecule signaling pathways (e.g., by receptor tyrosine kinases) [73]. ITG, integrin; RTK, receptor tyrosine kinase.

Increasing evidence suggests that oncogenes directly regulate the activity of adhesion molecules to control malignant hematopoietic progenitor cell proliferation, self-renewal, survival, and differentiation in cooperation with stromal cells within the bone marrow microenvironment. Dependencies on adhesion molecule function therefore represent “Achilles heels” for targeted treatment strategies. The frequency of detected mutations in adhesion molecules is, however, much lower compared with other molecules such as hyperactivated kinases, thereby suggesting that adhesion molecules do not directly exert oncogenic activity. In a few cases, the deciphered mechanisms of regulation suggest that oncogenic deregulation of cell adhesion can take place at several levels in the adhesion molecule-signaling cascade. Described and hypothesized mechanisms of CAM regulation include proteasomal degradation, activation by co-receptors, interference with composition of the adhesion molecule complex, and superactivation of pre-activated adhesion molecule pathways (Figure 4). Several oncogenes activate adhesion molecules such as integrins and selectins, which are critically involved in interaction with bone marrow niche cells. However, it remains unclear whether oncogene-mediated dysregulation of a particular adhesion molecule pathway represents a disease progression factor in every case. As mentioned, BCR-ABL1-induced *PSGL-1* downregulation dampens leukemic cell engraftment in CML and presumably does not accelerate the BCR-ABL1-induced transformation process. These observations suggest that, in certain cases, adhesion molecule regulation might be accidentally deregulated without providing growth advantage for the leukemic progeny. Therefore, the role that oncogene-mediated adhesion molecule regulation plays in leukemia development requires further investigation.

## Figures and Tables

**Table 1:** Detected somatic mutations of selected tyrosine kinases (TKs) and cell adhesion molecules (CAMs) typically expressed in hematopoietic stem and progenitor cells. The depicted genes were analyzed using the COSMIC database software (http://cancer.sanger.ac.uk/cancergenome/projects/cosmic/).

TKs		CAMs	
Gene	Mutant Samples	Gene	Mutant Samples
*c-KIT*	9.073	*PSGL1*	140
*FLT3*	17.467	*CXCR4*	472
*JAK2*	50.613	*CD34*	119
*ABL1*	1.961	*ITGα4*	430
